# Parental stress and support of parents of children with spina bifida in Uganda

**DOI:** 10.4102/ajod.v5i1.225

**Published:** 2016-05-31

**Authors:** Femke Bannink, Richard Idro, Geert van Hove

**Affiliations:** 1 Faculty of Psychology and Educational Sciences, Ghent University, Uganda; 2 Department of Paediatrics and Child Health, College of Health Sciences, Makerere University, Uganda

## Abstract

**Background:**

Children with disabilities in Sub-Saharan Africa depend for a large part of their functioning on their parent or caregiver. This study explores parental stress and support of parents of children with spina bifida in Uganda.

**Objectives:**

The study aimed to explore perceived stress and support of parents of children with spina bifida living in Uganda and the factors that influence them.

**Methods:**

A total of 134 parents were interviewed. Focus group discussions were held with four parent support groups in four different regions within the country. The Vineland Adaptive Behaviour Scales, Daily Functioning Subscales and Parental Stress Index Short Form (PSI/SF) were administered to measure the child’s daily functioning level and parental stress levels.

**Results:**

Parental stress was high in our study population with over half of the parents having a > 90% percentile score on the PSI/SF. Stress outcomes were related to the ability to walk (Spearman’s correlation coefficient [*ρ*] = −0.245), continence (*ρ* = −0.182), use of clean intermittent catheterisation (ρ = −0.181) and bowel management (*ρ* = −0.213), receiving rehabilitative care (*ρ* = −0.211), household income (*ρ* = −0.178), geographical region (*ρ* = −0.203) and having support from another parent in taking care of the child (*ρ* = −0.234). Linear regression showed parental stress was mostly explained by the child’s inability to walk (*β* = −0.248), practicing bowel management (*β* = −0.468) and having another adult to provide support in caring for the child (*β* = −0.228). Parents in northern Uganda had significantly higher scores compared to parents in other regions (Parental Distress, *F* = 5.467*; Parent–Child Dysfunctional Interaction, *F* = 8.815**; Difficult Child score, *F* = 10.489**).

**Conclusion:**

Parents of children with spina bifida experience high levels of stress. To reduce this stress, rehabilitation services should focus on improving mobility. Advocacy to reduce stigmatisation and peer support networks also need to be strengthened and developed.

## Introduction

### Spina bifida in Uganda

Spina bifida is a neural tube defect, a congenital abnormality causing disability, whereby the spinal cord and vertebrae do not form completely and the neural tube fails to develop normally. The worldwide incidence of spina bifida varies between 0.17 and 6.39 per 1000 live births (Bowman, Boshnjaku & Mclone 2009; Kinasha & Manji 2002; Msamati, Igbigbi & Chisi 2000; Shaer *et al*. 2007). In Uganda, incidence data are not available. Warf *et al*. estimated a birth incidence of 1 in 1000, translating into 1400 children born with spina bifida in the country annually (Warf *et al*. 2011).

Most children with spina bifida have some degree of paralysis, which affects mobility as well as bowel and bladder control (Northrup & Volcik 2000; Verpoorten & Buyse 2008). This affects participation in daily activities (Danielsson *et al*. 2008; Jansen *et al*. 2009). Sixty-six per cent of the children with spina bifida develop hydrocephalus (Warf & Campbell 2008).

Surgery and rehabilitative care are expensive and inaccessible for many born with the disability in Africa. Families need to find resources for children with disabilities whilst already constrained. In Uganda, the initial surgery (closure of the spine) at the time of this study was only available in Mulago National Referral Hospital in Kampala, and CURE Children’s Hospital in Mbale, eastern Uganda. Recently, Mbarara Regional Referral Hospital started offering the same. In north, west and central Uganda, three rehabilitation centres offer specific occupational therapy, physiotherapy, continence management and social support services for children with spina bifida and their families in Uganda (Supplement Figure 1: map). Policies such as the United Nations Convention on the Rights of Persons with Disabilities and Disability Act, amongst others, are in place and efforts are made by the Ugandan government and not-for-profit organisations and hospitals to provide basic services for children with *spina bifida* and other disabilities (Mertens & Bannink 2012; Oyaro 2014). Despite these efforts, poverty continues to affect access to services for the majority of the population and more so for the families of children with disabilities (Bannink *et al*. 2015; Lwanga-Ntale 2003; Miles 2002).

**FIGURE 1:**
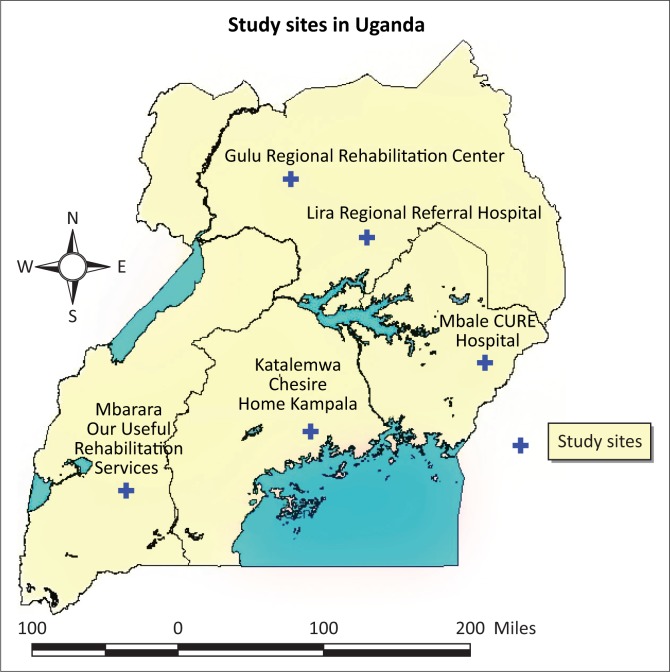
Map of geographical area.

### Parental stress and disability

Parental functioning is of great importance for children with severe disabilities who depend for a large part of their functioning on the parent or caregiver. Research on family functioning and psychosocial adjustment of families of children with spina bifida in high-income countries support a resilience–disruption view of family functioning, whereby the presence of a child with spina bifida disrupts normative family functioning at first, but after a period of time families adapt and exhibit considerable resilience (Holmbeck *et al*. 1997; Vermaes *et al*. 2007). The level of impairment has not been related to the general level of family functioning in Europe and the United States (Ulus *et al*. 2012; Wiegner & Donders 2000). Nevertheless, spina bifida has been found to have negative medium–large effects on parents’ psychological adjustment and functioning (Holmbeck & Devine 2010; Vermaes *et al*. 2005, 2008).

In the review by Vermaes *et al*. (2005), child, parent, family and environmental factors were found to be associated with variations in parents’ psychological adjustment. Mothers are often at higher risk for parenting stress than fathers because of role differences in care and work (Vermaes *et al*. 2008). Children with hydrocephalus (literally ‘excess water in the brain’) are often in need of neurosurgical treatment, which may involve insertion of a shunt or creation of a bypass within the brain to allow the cerebrospinal fluid drain. Shunts are prone to infections, which can be life threatening. For children with both spina bifida and hydrocephalus, such shunt dependency has been associated with higher levels of parental anxiety and depression (Malm-Buatsi *et al*. 2015).

Cultural differences have been described in a study of Hispanic parents in the United States, in which mothers of children with spina bifida were found to be at risk for lower feelings of satisfaction and competence as parents compared to non-Hispanic parents (Devine *et al*. 2012). In Malaysia, mothers of children with spina bifida had significantly higher stress scores compared to mothers with children without a disability (Ong *et al*. 2011). In the same country, clean intermittent catheterisation was the only medical factor associated with such stress. This is a technique used to empty urine from the bladder using a catheter. Most children with myelomeningocele, the severe type of spina bifida in children involved in this study, are incontinent of urine and benefit from practicing intermittent bladder catheterisation as this helps them to participate in daily activities without losing urine (IFSBH 2014). Parental stress in Malaysia was also mediated by single parenthood, caregiver status and the child’s adaptive skills (Kanaheswari *et al*. 2011).

African studies on parental stress in parents of children with spina bifida are limited. In South Africa, Greeff and Nolting showed that families of children with developmental disabilities adapt better when they are more accepting of the situation, have more investment in the family unit and have positive patterns of communication and attitudes towards challenges (Greeff & Nolting 2013). In Nigeria and Tunisia, parents of children with neurodevelopmental disabilities and learning disabilities had higher rates of depression and anxiety compared to parents of children without a disability (Abasiubong, Obembe & Ekpo 2006; Ben Thabet *et al*. 2013). In Malawi, parents of children with neurodisability following brain injury experienced barriers to care and support (Paget *et al*. 2016). Similar findings were seen in South Africa in families of children with mental health disabilities (Coomer 2013). In Kenya, mothers of children with spina bifida struggled with the financial implications of the child’s disability; most of them received some support from other parents and religious communities (Vant Veer *et al*. 2008).

In the study, we aim to explore perceived stress and support of parents of children with spina bifida living in Uganda and examine the factors that influence it. By understanding the stress factors, we hope to inform health and community-based rehabilitation services for children and parents with spina bifida in the country. We believe parental stress and support is highly dependent on the cultural and socio-economic context where parents live. Contextual factors such as the differences in rural and urban setting, and the 22-year conflict between the Lord’s Resistance Army and the Government of Uganda, which displaced over 2 million people in the north of the country (Muyinda & Whyte 2011), will be taken into account.

## Research methods

### Ethical considerations

Ethical approval and research clearance for the study were obtained from Ghent University, Belgium, the Uganda Virus Research Institute and the Uganda National Council for Science and Technology. Informed consent was obtained from all parents.

### Recruitment of participants

Selection of participants was performed in Mbarara, Kampala and Mbale, where CURE holds bi-monthly clinics. CURE hospital and the partnering rehabilitation centres in Kampala and Mbarara were requested to list all children registered in their follow-up programmes and inform them during home visits and through telephone calls to attend the clinic. In Gulu and Lira where no follow-up system or registry of the children was in place at the time, radio announcements were aired to inform parents about the upcoming review clinics in the area, and specifically invited parents with children with spina bifida between the age of 4 and 14 to attend.

**Data collection**

Explorative mixed-study methods were employed to explore factors affecting parental stress and support in the Ugandan setting (Creswell & Clark 2007) (Singh 2007). Both quantitative and qualitative data were collected concurrently from 134 parents between June 2011 and December 2014.

Quantitative instruments were used to explore daily functioning and stress levels. A selection of 10 items of The Vineland Adaptive Behaviour Scales (VABS) Daily Living Skills subscale relevant to the Ugandan setting were used. Items included measures of daily functioning tasks such as removing a jumper, drinking from a cup, washing ones’ face, fetching water and dressing independently. Items were scored 2 (behaviour is usually or habitually performed), 1 (sometimes or partly performed) or 0 (never performed) (Sparrow *et al*. 1984).

The Parental Stress Index Short Form (PSI/SF) consists of 36 items scored on a 5-point Likert scale. The items are divided over three subscales: parental distress (PD), parent–child dysfunctional interaction (P-CDI) and difficult child (DC). A total stress score is computed from the three subscales and indicates the overall level of parenting stress in the areas of personal PD, stresses derived from the parent’s interaction with the child and stresses that result from the child’s behavioural characteristics. Parents who obtain a total stress score above 90 are experiencing clinically significant levels of stress (Abidin 1995).

The presence of a (house) helper or other adults at home involved in the care of the child alongside the primary caregiver was registered as a measure of perceived support. Membership of a parent support group was documented to understand wider support networks.

Qualitative methods were used to explore the day-to-day reality of parents of children with spina bifida in the Ugandan setting. Semi-structured interviews were held with all 134 parents. In addition, focus group discussions were held with four parents’ support groups, in Gulu, Kampala, Mbale and Mbarara, respectively. Questions for the interviews and focus group discussions were formulated to develop a more in-depth understanding of perceived parental stress, coping strategies and the role of support from others. Specific focus group discussions with parent support groups were held to further understand their activities and possible contributions in reducing parental stress and increasing perceived support. In total 5 of the 134 parents took part in the focus group discussions, the other members of the focus groups (26) were not interviewed individually.

The interviews with the parents were held in the local language of the area, and a translator was hired and trained for each area to assist in conducting the interviews and focus group discussions. Some of the interviews were conducted in English, if parents were fluent and requested for this.

### Data analysis

The semi-structured interviews and focus group discussions were transcribed, translated, coded and analysed using thematic analysis in NVivo version 10. Basic demographic data, details on the child’s impairment, social support and scores for the VABS Daily Functioning Subscale and PSI/SF were entered and analysed using SPSS16. The sub-total scores for each subscale was calculated and compared with > 90% percentile scores from the PSI manual, which indicates high levels of stress. To understand relationships between demographic and impairment variables and parental stress levels, Spearman’s rho correlations were computed. To compare regional differences, a multivariate analysis of variance (MANOVA) between PSI scores between the various regions was carried out. Linear regression analysis was conducted to understand the factors that mostly contributed to parental stress.

## Results

### Description of study participants

Table 1 describes the demographics of the study population. Parents’ ages ranged from 24 to 46 years with an average age of 32.9 (SD 5.2) years. The majority of the parental respondents were the mother to the child, followed by fathers and grandmothers. Over 75% was married and Christian. Almost half of them were farmers.

**TABLE 1:** Demographics of the study population (*n* = 134).

Demographic variables	Number	Percentage
**Caregiver relationship**		
Mother	104	77.6
Father	15	11.2
Grandmother	9	6.7
Other	6	4.5
**Education level of caregiver**		
None	6	4.7
Primary	72	56.2
Secondary	28	21.9
Vocational	11	8.6
University	11	8.6
**Marital status of caregiver**		
Single	12	8.8
Married	100	75.2
Separated	9	8.0
Widowed	11	8.0
**Occupation of parent**		
Finance/administration	5	3.8
Small-scale private business	30	22.6
Teacher/education	9	6.8
Medical/paramedical	5	3.8
Civil service/government	3	2.3
Peasant farmer	65	48.9
No occupation	16	12.0
**Child is schooling in**		
Nursery school	49	37.4
Primary school	22	16.8
Secondary school	4	3.1
Not schooling	56	42.7
**Type of disability of the child**		
Spina bifida	73	54.5
Spina bifida and hydrocephalus	61	45.5
**Location/region**		
Central	63	47.0
East	23	17.2
West	29	21.6
North	19	14.2
**Religion**		
Christian	101	77.3
Muslim	26	19.4
Other	3	2.3
**Monthly household income**		
< 30 euros	25	19.5
30–60 euros	28	21.9
61–90 euros	26	20.3
> 90 euros	49	38.3

The mean age of the 80 (59.7%) male and 54 (40.3%) female children with spina bifida (myelomeningocele type) was 6.1 (SD = 2.0) years, ranging from 4.0 to 14.0 years of age. Only 76 (57.3%) of them were going to school: 49 (37.4%) were in nursery school, 22 (16.8%) in primary school and 4 (3.1%) in secondary school. The household size ranged from 2 to 13 with an average of 6.5 persons per household; an average 4.3 children (SD 2.2) and 2.3 adults (SD 1.0) per household. The average monthly household income is 82 euros (range, 12–604 euros). The majority of the children (128, 95.5%) had undergone surgery to close their spine (myelomeningocele closure) earlier in life. Of the 61 children who had both spina bifida and hydrocephalus, 22 (35.9%) had undergone endoscopic third ventriculostomy whilst 1 (18.6%) had ventriculo-peritoneal shunts placed. Only 2 (3.6%) of the children of parents in the study never had surgery. Most parents took their children (118, 90.8%) for rehabilitation services such as physiotherapy and occupational therapy.

### Parents’ stress index outcomes

The parent stress index questionnaire was administered to all parents. Scores were normally distributed for all items including the total subscales. We excluded three cases from the analysis as scores showed defensive responding (score < 10). Table 2 shows the subscales scores. More than half of the parents (52.7%) scored above the 90% percentile on total stress. Higher scores were seen on the P-CDI domain with 48.1% (63) scoring above the 90% percentile, followed by the DC Domain with 32.8% (43) scoring above the 90% percentile and the PD Score in which 14.5% (19) of the parents scored above the 90% percentile.

**TABLE 2:** PSI/SF subscale and total scores for 131 parents of children with spina bifida.

PSI/SF domain	Range	Mean (SD)	High score (> 90% percentile), *n* (%)
PD score	14–52	28.4 (10.0)	19 (14.5)
P-CDI score	14–55	30.1 (8.7)	63 (48.1)
DC score	14–50	31.6 (8.3)	43 (32.8)

**Total score**	**50–153**	**90.2 (23.9)**	**69 (52.7)**

PD, Parental Distress; P-CDI, Parent Child Dysfunctional Interaction; DC, Difficult Child.

Spearman’s rho correlations were calculated for demographic variables, impairment-related variables, for example incontinence and mobility, daily functioning skills and perceived support from another adult and parent support groups. Table 3 shows the variables that had significant Spearman’s rho correlations. Variables with significant correlations with PSI outcomes were the child’s ability to walk, continence and use of clean intermittent catheterisation and bowel management, receiving rehabilitative care, household income, location (region where the child and family lives) and having support from another parent in taking care of the child. Household income correlated positively with school going, region, parental education, the use of assistive devices, receiving rehabilitation services, using catheterisation and having support from another adult.

**TABLE 3:** Spearman’s rho correlations PSI/SF scales (***n*** = 134)

Variable	PSI	PSI PD	PSI P-CDI	PSICD	Gender	Child is schooling	Location	Household income	Relation ship parent child	Education of parent	Daily functioning VABS	Hydroce phalus	Able to walk	Assistive devices	Rehabilitation received	Continent urine	Uses Clean Intermittent Catheterisation	Continent stool	Uses bowel management	Parent has support of an adult	Parent is a member of a PSG
PSI total score	1	0.758‡	0.748‡	0.752‡	-0.049	0.033	0.203†	-0.105	-0.004	-0.14	-0.136	0.171†	-0.254‡	0.091	-0.211†	0.103	-0.181†	0.026	-0.213†	-0.119	0.032
PSI PD	0.758‡	1	0.713‡	0.653‡	-0.029	0.101	0.298‡	-0.178†	-0.022	-0.152	0.055	0.077	-0.180†	0.202	-0.154	0.063	-0.095	0.021	-0.167	-0.169	0.065
PSI P-CDI	0.748‡	0.713‡	1	0.715‡	-0.005	-0.006	0.313‡	-0.142	-0.037	-0.182†	-0.102	0.145	-0.200†	0.041	-0.079	0.182†	-0.168	0.146	-0.185†	-0.234‡	0.056
PSI DC	0.752‡	0.653‡	0.715‡	1	-0.092	-0.013	0.220†	-0.162	0.022	-0.158	-0.022	0.13	-0.231‡	-0.008	-0.173†	0.114	-0.202†	0.069	-0.204†	-0.096	0.003
Gender	-0.049	-0.029	-0.005	-0.092	1	-0.091	-0.081	-0.05	0.186†	-0.03	0.083	0.043	0.126	0.126	0.002	-0.001	0.146	-0.01	0.125	0.069	-0.041
Child is schooling	0.033	0.101	-0.006	-0.013	-0.091	1	-0.231‡	0.199†	0.006	0.198†	0.400‡	-0.055	0.253‡	0.368‡	0.158	0.220†	0.331‡	0.172	0.239†	0.106	0.122
Location	0.203†	0.298‡	0.313‡	0.220†	-0.081	-0.231‡	1	-0.269‡	-0.144	-0.340‡	-0.031	0.108	-0.171	-0.08	-0.129	-0.026	-0.171	-0.02	-0.181	-0.114	0.277‡
Household income	-0.105	-0.178†	-0.142	-0.162	-0.05	0.199†	-0.269‡	1	0.004	0.373‡	-0.002	-0.051	-0.044	0.499‡	0.229‡	0.014	0.218†	-0.071	0.172	0.263‡	-0.027
Relation ship parent child	-0.004	-0.022	-0.037	0.022	0.186†	0.006	-0.144	0.004	1	-0.119	0.024	0.024	-0.008	-0.026	0.188†	-0.148	0.183†	-0.153	0.16	-0.006	-0.185†
Education of parent	-0.14	-0.152	-0.182†	-0.158	-0.03	0.198†	-0.340‡	0.373‡	-0.119	1	0.079	-0.027	0.013	0.061	0.233‡	0.159	0.181	0.186†	0.161	0.039	-0.008
Marital status	-0.036	-0.076	-0.114	0.028	-0.078	0.166	-0.112	0.285‡	0.169	0.129	0.015	0.028	-0.094	0.223	0.105	-0.024	0.132	0.015	0.141	0.092	0.001
Daily functioning	-0.136	0.055	-0.102	-0.022	0.083	0.400‡	-0.031	-0.002	0.024	0.079	1	-0.052	0.507‡	0.265	0.219†	0.184†	0.243‡	0.167	0.165	0.078	0.133
Hydrocephalus	0.171†	0.077	0.145	0.13	0.043	-0.055	0.108	-0.051	0.024	-0.027	-0.052	1	-0.255‡	-0.066	-0.083	0.051	-0.095	-0.014	-0.099	-0.066	0.128
Able to walk	-0.254‡	-0.180†	-0.200†	-0.231‡	0.126	0.253‡	-0.171	-0.044	-0.008	0.013	0.507‡	-0.255‡	1	-	0.172	0.185†	0.233†	0.224†	0.151	0.015	0.069
Assistive devices	0.091	0.202	0.041	-0.008	0.126	0.368‡	-0.08	0.499‡	-0.026	0.061	0.265	-0.066	-	1	0.339†	0.113	0.455‡	-0.036	0.434‡	0.224	0.245
Rehabilitation received	-0.211†	-0.154	-0.079	-0.173†	0.002	0.158	-0.129	0.229‡	0.188†	0.233‡	0.219†	-0.083	0.172	0.339†	1	0.012	0.505‡	0.03	0.475‡	0.044	0.124
Continent urine	0.103	0.063	0.182†	0.114	-0.001	0.220†	-0.026	0.014	-0.148	0.159	0.184†	0.051	0.185†	0.113	0.012	1	-0.006	0.762‡	-0.131	0.041	0.137
Uses CIC	-0.181†	-0.095	-0.168	-0.202†	0.146	0.331‡	-0.171	0.218†	0.183†	0.181	0.243‡	-0.095	0.233†	0.455‡	0.505‡	-0.006	1	0.043	0.896‡	0.113	0.213†
Continent stool	0.026	0.021	0.146	0.069	-0.01	0.172	-0.02	-0.071	-0.153	0.186†	0.167	-0.014	0.224†	-0.036	0.03	0.762‡	0.043	1	-0.092	-0.026	0.116
Uses bowel management	-0.213†	-0.167	-0.185†	-0.204†	0.125	0.239†	-0.181	0.172	0.16	0.161	0.165	-0.099	0.151	0.434‡	0.475‡	-0.131	0.896‡	-0.092	1	0.163	0.207†
Parent has support	-0.119	-0.169	-0.234‡	-0.096	0.069	0.106	-0.114	0.263‡	-0.006	0.039	0.078	-0.066	0.015	0.224	0.044	0.041	0.113	-0.026	0.163	1	0.023
Parent is PSG member	0.032	0.065	0.056	0.003	-0.041	0.122	0.277‡	-0.027	-0.185†	-0.008	0.133	0.128	0.069	0.245	0.124	0.137	0.213†	0.116	0.207†	0.023	1

† Correlation is significant at the 0.05 level (two-tailed);

‡ Correlation is significant at the 0.01 level (two-tailed).

Based on the significant correlations found, linear regression analysis was performed to investigate factors that were determinants for high scores in the PD, DC and P-CDI subdomains of the PSI/SF.

All variables with significant correlations were entered in a stepwise regression model. The best predictive models are displayed in Table 4. Results are expressed as beta coefficients, *R*^2^ values and *F* values in Table 4. The ability to walk, having support of another adult and the location where the child and family are residing were the significant contributing predictors.

**TABLE 4:** Linear regression model of PSI/SF total and subscale scores, with demographic, impairment and support-related predictors presenting (only significant predictors for each dependant variable).

Dependent variable	Significant predictors	Beta	*R^2^*	*F*
PD	Ability to walk *a* Bowel management *b*	-0.190† -0.468†	0.100	2.254†
P-CDI	Support of another adult *c* Location *d*	-0.228† 0.284‡	0.184	7.793‡
DC	Ability to walk Location	-0.218‡ 0.260‡	0.154	7.793†
Total parental stress	Ability to walk	-0.248†	0.109	3.114†

Linear regression analysis codes: *a*, child is able to walk unaided is 1, unable to walk unaided is 0; *b*, practising bowel management is 1, not practising is 0; *c*, having support is 1, not having support is 0; *d*, location north is 1, other is 0.

PD, Parental Distress; P-CDI, Parent Child Dysfunctional Interaction; DC, Difficult Child.

† < 0.05;

‡ < 0.01.

MANOVA results showed that parents in northern Uganda had significantly higher > 90 percentile scores on the PSI subscales compared to parents in other regions: PD *F* = 5.467*, P-CDI *F* = 8.815** and DC *F* = 10.489**. No significant differences were found between the other regions.

### Children’s mobility and incontinence

The inability to walk was the largest contributor to parental stress and perceived parental difficulty in managing and caring for the child. Parents of children with both spina bifida and hydrocephalus had higher scores on dysfunctional interaction between parent and child compared to those with only spina bifida only.

The majority of the children with spina bifida whose parents were interviewed were able to sit without assistance and could speak. Half of them were able to walk without assistive devices. Of the ones who were in need of assistive devices, a third used crutches, another third a wheelchair, whilst the others had no access to assistive devices and crawled. In our study population, 88.6% of the children were incontinent and 81.1% of them used clean intermittent catheterisation to manage the incontinence. Table 5 shows the various percentages for the impairment-related variables.

**TABLE 5:** Children’s mobility and physical impairments.

Impairment variable	Yes	No
Child can sit without assistance	131 (97.8%)	3 (2.2%)
Child is able to speak	127 (94.8%)	7 (5.2%)
Child is able to walk without assistive devices	62 (49.2%)	64 (50.8%)
Child is continent of urine	15 (11.4%)	117 (88.6%)
Child uses clean intermittent catheterisation	99 (81.1%)†	23 (18.9%)
Child is continent of stool	18 (13.4%)	114 (86.4%)
Child uses bowel management	91 (78.4%)‡	25 (21.6%)

†, 23 (35.9%) use a wheelchair, 23 (35.9%) use crutches, 17 (28.1%) crawl and 22 (16.4%) practice Clean Intermittent Catheterisation (CIC) without the assistance of another person.

‡, 7 (6.9%) practice bowel management without the assistance of another person.

In the interviews, parents indicated mobility was a major challenge and stressor. Many parents narrated how they carried their children on their back to the land they cultivate (most of them are subsistence farmers), to public transport and sometimes to school when the children were young. Some still continue to do so as assistive devices are not always available or applicable for use in their setting.

‘She is getting heavy now. She was easy to carry, she is still a bit small, but her head is heavy. I have no alternative; there is no one to help at home, so I have to bring her with me to the garden when I go to dig. I put her under a tree in the shade.’ (Parent of a 5-year-old girl with spina bifida in northern Uganda)

‘When it is rainy season our roads are too slippery for him. He can’t go to school then. On other days the wheelchair is good, we can push him, and he tries to use it himself.’ (Parent of an 11-year-old boy with spina bifida in western Uganda)

Although incontinence did not explain parental stress for a large part on the PSI outcomes, apart from practising bowel management on the PD subscale, in the focus group discussions and interviews the majority of parents said incontinence was the most complex factor in managing their child’s health, more than the (partial) paralysis. Whilst the majority did practice catheterisation, parents complained practicing catheterisation and bowel management outside home and the rehabilitation posed a big challenge. Managing their children’s catheterisation meant that parents had to be close by and available to help the child practice catheterisation, for example at school every 6 hours. Accidents or ill-managed catheterisation also caused stress as the child would be wet and smelly, which would result in others commenting or pitying the parents.

‘We have used catheterization since my child was 2 years old. We are used to it, but when we go out or I am away, it is difficult. In most public places there is no place to practice catheterization. We have to do this behind the latrine in the open, which is not good.’ (Parent of a 4-year-old daughter with spina bifida and hydrocephalus in western Uganda)

### Parents’ support systems

Almost half of the parents (63, 47.7%) mentioned they have support of another adult in the household to care for their child with spina bifida. Having another adult to support in providing care significantly contributed to having less dysfunctional parent–child interaction on the PSI outcomes. This other adult would be a spouse, relative or house help. In the focus group discussion and interviews, parents who did not have support from another adult explained they found it very stressful to be constantly responsible for their child and often felt isolated, as they could not attend family and community functions because of their care obligations. Whilst some could leave their other children with neighbours or friends, they said it was hard to find someone who would be ready to take care of their child with spina bifida. Parents in the north and east had less support from other adults compared to those in the central and western regions.

‘I don’t have a house help, when I need to go somewhere I have to take her with me. I can’t leave her with the neighbours; they don’t know how to look after her.’ (Parent of an 8-year-old daughter with spina bifida in eastern Uganda)

In total, 55 (41%) parents were members of a parent support group. No interaction was found between PSI outcomes and membership of a parent support group. Table 6 provides details of the reasons of (not) participating in a parent support group. From the interview, those who were members of a parent support group indicated it mostly helped them to learn more about taking care of their child (50.9%), followed by feeling encouraged by other parents (14.5%), enjoying sharing experiences and learning from each other (14.5%) and income-generating opportunities (12.7%).

‘Coming to the parent support group is helpful because they teach me how to do catheterization and they understand my problems.’ (Parent of a 4-year-old girl with spina bifida in the western region)

**TABLE 6:** Reasons for (not) participating in parents support groups for parents of children with spina bifida and hydrocephalus.

Benefits of participating in Parents Support Group (*n* = 56)	Number	Frequency (%)
Learning about taking care of child with spina bifida	28	50.9
Encouragement from other parents	8	14.5
Sharing experiences, learning from each other	8	14.5
Learning how to include my child in school	1	1.8
Income-generating activities/opportunities	7	12.7
Not sure, just joined	2	3.6
Reason unknown	2	3.6
**Reason for not participating in Parents Support Group (*n* = 78)**		
Not aware of existence of parents support group	43	55.1
Not in their location/far away	23	29.5
Is planning to join, was not aware	5	6.4
Does not have time	3	3.9
Reason unknown	4	5.1

The majority of parents who were not members of a parent support group said they were not aware parent support groups existed (55.1%), and some mentioned they had heard about parent support groups but lived too far away from the rehabilitation centre to attend meetings regularly.

‘I did not know there is a support group […] Also I live far away, I travel 3 hours to Gulu [*where the PSG meets*]. It is not easy to come.’ (Parent of a 7-year-old boy with spina bifida in the northern region)

To explore the support given by the PSG further, focus group discussions were held with 26 mothers and 6 fathers active in parents support groups in Gulu (9: 8 mothers, 1 father), Kampala (6: 5 mothers, 1 father), Mbale (8: 6 mothers, 2 fathers) and Mbarara (9: 7 mothers, 2 fathers).

Parents explained the groups started with help of the rehabilitation centres in three sites; in Gulu, the group started based on a community-based rehabilitation initiative which was started up during the conflict in the area. The main objectives of the groups were to motivate and support each other in caring for their children, provide information and training on care and rehabilitation and create awareness in their communities about the disability. Parents said they themselves enjoyed participating in the PSG as they would learn more about taking care of their child and were able to advise and encourage others. The majority indicated that the feeling of ‘not being alone’ in this situation and being able to share specific challenges with others with similar experiences helped them to continue caring for and loving their child more.

‘When I attended the first time, I was so surprised and happy I was not the only one with a child like mine. There were children who could not even sit. The other parents were so encouraging; they gave me hope and a lot of information and ideas on how to help my child and myself.’ (Parent of a 7-year-old daughter with spina bifida and hydrocephalus in central Uganda)

Some parents mentioned the group had given them new friends and support networks. For example, in case of advocating for children, parents in Kampala and Mbarara gave examples on how other parents would join them in visiting schools trying to get a space for their child. Many schools would refuse children with physical impairments, but if going as a group or pair, head teachers were said to take their call more seriously and were more likely to give them a space. Similar advocacy took place on inclusion and reducing stigma in communities, where parents would move around with their children explaining the impairment to community members and the need for their children and families to be included. In Mbale, most members felt the group gave them spiritual support too.

‘I had gone to 4 schools and every time the head teacher said they can’t manage my child in school. There was a parent in the group, she had the same problem but had managed to find a school. She was a very strong woman. She came with me and spoke with the head teacher of a school near our home. He accepted and now my child is in primary 1.’ (Parent of an 8-year-old daughter with spina bifida in western Uganda)

‘The PSG helps you to remember to come for follow-up and to work hard. It helps to talk with other parents and to know how to do catheterization. We pray for each other and thank God for our children and we tell others about them so they stop discriminating us.’ (Parent of an 11–year-old daughter with spina bifida in eastern Uganda)

In northern Uganda, most parents felt the group gave them an opportunity to start income-generating activities together. They had started a rotating loan scheme, which was helping members to set up small businesses. They especially felt stigma was high in their area, and support for them was difficult as some people believed the impairment was contagious. Similar activities were mentioned in Mbarara and Kampala. Stigma was mentioned in these areas too, but less directly connected to physical contact as in the northern region.

‘Here [*in the north*] people think our children’s disability is contagious, they fear us, they avoid us. In the PSG we can work together, we understand each other. We bought goats for the group, and when they produced we all got one. We started planting maize in the last rainy season together too.’ (Parent of a 4-year-old son with spina bifida and hydrocephalus in northern Uganda)

## Discussion

This study describes parental stress and support of parents of children with spina bifida in Uganda. PSI scores were high for our study population with more than half scoring above the PSI/SF cut-off point for clinically significant levels of stress (Abidin 1995). Stress outcomes were related to the inability to walk, continence and use of clean intermittent catheterisation and bowel management, receiving rehabilitative care, household income, region and having support from another parent in taking care of the child. Parental stress was mostly explained by the child’s inability to walk, practicing bowel management and having another adult to provide support in caring for the child.

Our study does not support the view that the level of impairment is not related to the general level of functioning or severity of the disability as earlier explained in studies of families with a child with spina bifida in Europe and the United States (Ulus *et al*. 2012) (Wiegner & Donders 2000). Not being able to walk has great implications in an environment in which assistive devices are not easily accessible and where the environment is disabling. There are no social services or individual disability grants provided by the Government of Uganda to families with children with disabilities. As narrated by parents in the qualitative data, parents are responsible for carrying their children to school and feel that even if a child has a wheelchair, they often cannot use this on their own. Earlier we showed how children with spina bifida had more difficulties in daily functioning than their siblings, which played an important factor in inclusion (Bannink, Idro & Van Hove 2016).

Parents expressed stress around incontinence management. Because of the high and frequent involvement of parents on a daily basis, this could increase stress to parents, the majority of whom are either farming or working and not in a position to attend to their child every 4–6 hours as required for clean intermittent catheterisation. Prior to self-catheterisation, which can be started from the age of 6 with normal intelligence and manipulative skills, parents are the primary helpers in practicing catheterisation (Robinson *et al*. 1985). The responsibility and anxiety around incontinence has a social and psychological impact on families (Borzyskowski *et al*. 2004). In our study, parents pointed out that catheterisation interfered with their lives, as they sometimes had to make extra trips to school to assist in catheterisation. Similar concerns were raised in the study by Kanaheswari *et al*. (2011) in Malaysia.

In our study, parents in the northern region expressed higher stress levels than those in other areas. Northern Uganda suffered a 22-year-long conflict between the Government of Uganda and the Lord’s Resistance Army in which 90% of the population was displaced (Muyinda & Whyte 2011). Together with challenges related to recovery from this conflict, parents in this area face more stigmatisation because of negative cultural beliefs about the impairment (Bannink *et al*. 2015). This is likely to increase stress and have more need for support from other parents as expressed in the interview and focus group discussion findings.

We did not find differences between mothers and other caregivers in the stress levels. Mothers are often at higher risk for parenting stress than fathers because of role differentiations in care and work (Vermaes *et al*. 2008). Kanaheswari (2011) found that mothers who were the sole caregiver had higher parenting stress scores in the P-CDI domain (Kanaheswari *et al*. 2011).

In our study, we did find that having support from another adult contributed to less stress. Support here was described by parents as household support and caregiving support at home, for example through a house help or relative. This support reduced dysfunctional interaction between parent and child. Parents who were a member of a parent support group felt this helped them in taking better care of their child and felt encouragement from other parents.

Persons with disabilities typically live in poorer than average households and have lower educational attainment (Emmett 2006; Filmer 2008; Lwanga-Ntale 2003). The monthly income of families in our study ranged from $28 - $689 with a median of $82 (income derived from all sources includes wages, market sells, cattle, land and other assets). This is much lower than the total national average of $156 (converted from Ugandan Shillings), although closer to the average rural income of $112 and regional variations (Uganda Bureau of Statistics 2014).

Although no significant effect was seen on parental stress in the regression analysis, looking after a child with spina bifida raises financial costs for families in terms of medical treatment, rehabilitation and transport. Correlations were found between having higher income and school going, the use of assistive devices, receiving rehabilitation services, using catheterisation and having support from another adult. Whilst each of these factors on their own may not contribute to parental stress, parents with higher household income are expected to be able to respond to their children’s medical needs better than those who have less. Parents who participated in parent support groups indicated that they benefitted from income-generating activities that the group organised.

We noted that less than half of the participants’ children had both spina bifida and hydrocephalus. Given the earlier established rates of 66% hydrocephalus in children with spina bifida (Warf *et al*. 2011), this is low. We suspect that more children with hydrocephalus died compared to children with only spina bifida, as shunt complications and failure is high and life threatening, especially in rural areas. Whilst Warf *et al*. (2011) found no relationship between survival and presence of hydrocephalus, we suspect this may have an effect for the older group of children targeted in this study, especially those living in hard-to-reach areas (Warf *et al*. 2011).

Participation in parent support groups did not make significant changes in parental perceived stress in the quantitative outcomes. However, from the qualitative outcomes, we see that the sense of belonging and being able to share was an important support for parents and enabled them to cope with stress better. Malm-Buatsi *et al*. (2015) found that involvement in recreational activities with other families affected by spina bifida was associated with more positive parenting characteristics. Our study did not consider parenting styles, but did receive positive feedback from parents about improved interaction with their child after having participated in parent support groups. Lack of knowledge and acceptance of a child with spina bifida may lead to neglect. Parent support groups aim to raise awareness and support for parents to look after their children positively. Warf *et al*. (2011) described finding situations of child neglect during home visits in the eastern region.

This study was limited by involvement of parents of only those children who were receiving or are attending follow-up and rehabilitation care. This means we interviewed those who may have been doing ‘better’ than others, as we only found those whose children accessed surgical care and survived.

Although the impairment factors and support explained some of the parental stress, a large part remained unexplained in our study. We expect that a complex combination of child, parent, family and environmental factors may contribute to parental stress in a low-income setting. Vermaes *et al*. (2005) found that a combination of these factors explains parents’ psychological adjustment.

Further studies need to be conducted to understand the interaction between poverty, survival and functioning of children with spina bifida and their parents in low-resource settings. Participation in parent support groups could be a protective factor for the child, reducing child neglect. We suggest analysing this contribution in greater detail and tailoring interventions to raise awareness and to take prompt action of support networks and state actors to ensure child protection and care. Studies on parental characteristics and coping styles could help inform supportive activities further.

Within the Ugandan setting, we argue for more investment in community-based rehabilitation to improve mobility and care for children with spina bifida and their parents. Sensitisation, especially in areas where stigmatisation appears high such as the northern region, is recommended. We recommend continuous support and creation of peer support networks for parents from government and civil society. We argue for awareness raising on spina bifida to increase engagement of relatives, house helps and community members in the care of children with spina bifida. We believe this will help reduce parental stress and improve the quality of life of both children with spina bifida and their parents.

## Conclusion

Parents of children with spina bifida experience high levels of stress. The degree of such stress is partly influenced by the level of impairment and need for parental involvement in care, support received from other adults and area of residence of the family. To reduce the parental stress, rehabilitation services should focus on improving mobility, advocacy to reduce stigmatisation and strengthening in communities and developing peer support networks.
